# Editorial: Adipocyte differentiation, pluripotency and molecular mechanisms

**DOI:** 10.3389/fendo.2022.1037159

**Published:** 2022-09-26

**Authors:** Jin Young Huh, Damian G. Romero

**Affiliations:** ^1^ Institute of Molecular Biology and Genetics, School of Biological Sciences, Seoul National University, Seoul, South Korea; ^2^ Department of Cell and Molecular Biology, University of Mississippi Medical Center, Jackson, MS, United States; ^3^ Mississippi Center of Excellence in Perinatal Research, University of Mississippi Medical Center, Jackson, MS, United States; ^4^ Women’s Health Research Center, University of Mississippi Medical Center, Jackson, MS, United States; ^5^ Cardiovascular-Renal Research Center, University of Mississippi Medical Center, Jackson, MS, United States

**Keywords:** obesity, adipocyte differentiation, stem cell, inflammation, lipid metabolism

Adipose tissue is a critical organ for both conserving the extra energy and supplying it to other tissues as needed, such as in fasted conditions (Choe et al.; 1). However, obesity-mediated adipose tissue expansion undergoes various stress responses, such as inflammation, ER stress, and hypoxia, leading to insulin resistance, catecholamine resistance, or inflammatory responses. These stress responses dysregulate normal adipose tissue function as a lipid storage organ, which exacerbates ectopic fat accumulation. Also, adipose tissue has a significant role as an endocrine organ that releases various hormones known as adipokines to control systemic energy metabolism. Particularly, brown adipose tissue (BAT), which has a distinct property of energy consumption rather than energy storage, may be approached to ameliorate obesity and its related metabolic diseases through the prompting of thermogenesis. Thermogenesis in BAT is mainly mediated *via* uncoupling protein 1 (UCP1) localized in mitochondria. White adipocytes can also play a role in thermogenesis, *via* browning, differentiating into beige adipocytes.

This Research Topic covers recent groundbreaking research on the regulatory factors for adipocyte differentiation and energy metabolism, as well as the regulatory mechanisms of adipose-derived stem cells in the context of therapeutic targets for stress urinary incontinence (SUI). This Research Topic consists of seven original manuscripts and one mini-review.

The original manuscript by Montt-Guevara et al. reported that D-Chiro-Inositol (D-Chiro-Ins), one of the inositol stereoisomeric forms, could improve fat or glucose storage function by promoting the signaling of insulin and estrogen in adipocytes. They suggested that enhanced expression of IRS1 and GLUT4 upon D-Chiro-Ins treatment during differentiation can be exploited to increase lipid/glucose storage capacity in human adipocyte cell lines generated from Simpson-Golabi-Behmel syndrome.

The original manuscript by Bové et al. discusses how the neurotrophin-3 (NT3)/tropomyosin-related kinase receptor C (TrkC) pathway functions in adipocytes. NT3, known to transmit its intracellular signaling through TrkC, was described as a protein that mediates neuronal survival and synapse formation. Bové et al. discovered that NT3 expression is present in adipose tissue of both humans and rats, and its expression level declines with aging. TrkC was expressed in adipocytes, whereas NT3 was primarily found in blood vessels in adipocytes. Upon NT3 treatment, rat adipocyte differentiation showed suppressed differentiation with enhanced lipolysis and UCP1 expression. In addition, a mouse model with reduced expression of NT3 or endothelial cell-specific NT3 deficiency displayed larger adipocyte size with decreased UCP1 expression. Therefore, the authors concluded that NT3/TkrC axis would modulate adipocyte differentiation and browning.

The original manuscript by Zhao et al. investigated the roles of a secretable FDNC5 (sFDNC5), a precursor form of irisin which is known as an exercise-driven hormone, in adipocyte energy metabolism. Zhao et al. expressed and extracted sFDNC5 using a yeast expression system (*Pichia pastoris*) that can carry out eukaryotic posttranslational modification, including glycosylation, with high yield. Recombinant sFDNC5 stimulated the expression of thermogenic genes including UCP1 in 3T3-L1 adipocytes, whereas it reduced adipogenesis. Thus, the authors proposed recombinant sFDNC5 as a candidate target for the treatment of obesity and related metabolic diseases.

The original manuscript by Tang et al. suggests that angiopoietin-like protein 8 (ANGPTL8) can promote adipogenesis in mesenchymal stem cells. ANGPTL8 is a secretory factor mainly produced by liver and adipose tissues (2). In this manuscript, Tang et al. observed that high-fat diet (HFD)-induced gain of body weight and fat/liver mass was alleviated in ANGPTL8 knockout (KO) male mice. In particular, ectopic fat deposition in kidney, heart, and liver was reduced in KO mice. Interestingly, body weight reduction was not observed in female KO mice because estrogen inhibits the expression of ANGPTL8, a finding further proved by ovariectomy in female mice. Furthermore, the manuscript reports that ANGPTL8 promotes adipogenesis by inhibiting the Wnt/β-catenin signaling, and suggests that ANGPTL8 could be a novel therapeutic target for preventing or treating ectopic lipid accumulation.

The original manuscript by Xu et al. reports on the inhibitory effect of the selective phosphodiesterase 4 (PDE4) inhibitor Roflumilast on adipogenesis. PDE4 is an enzyme that breaks down cAMP, and it has been demonstrated that mice lacking the PDE4 enzyme have decreased fat mass (3). Xu et al. showed Roflumilast-stimulated AMPK activation in 3T3-L1 adipocytes and HFD-fed mice, which was accompanied by diminished adipogenesis and lipid accumulation with increased lipolysis. This finding raised the prospect that Roflumilast might be used to treat obesity by modulating energy metabolism.

The original manuscript by Petito et al. aimed to determine the effects of 3,5-diiodo-tyrosine (T2) on obesity-related adipose tissue inflammation. This study showed that T2 treatment improved insulin sensitivity and decreased fat mass in HFD-fed rats. In adipose tissue, proinflammatory response decreased concurrently with an increase in anti-inflammatory M2 macrophage gene expression. Serum levels of adiponectin, IL-13, IL-10, and IL-4 increased along with the downregulation of inflammatory cytokines such as TNF, IL-6, and PAI-1, which reveals the protective impact of T2 against obesity-induced inflammation.

The original manuscript by Wang et al. demonstrated the mechanism by which adipose-derived mesenchymal cells (ADSCs) exert a beneficial role in the treatment of stress urinary incontinence (SUI). This study suggests that ADSCs secrete extracellular vehicles (EVs) carrying microRNA-93 to inhibit coagulation factor III (F3) expression in fibroblast and satellite cells. Consequently, microRNA-93 can be involved in SUI improvement by altering extracellular matrix remodeling and activating satellite cells *via* regulating F3 expression.

The mini-review manuscript by Zhang et al. focused on the function of BAT in the context of Polycystic Ovary Syndrome (PCOS) therapy. The enhancement of BAT function is considered advantageous in metabolic diseases through BATokine secretion or energy consumption (4). PCOS patients are characterized by hyperinsulinemia and insulin resistance, representing the close linkage between PCOS and metabolic dysregulation (5). In this mini-review, Zhang et al. discussed the impact of BAT activation through cold exposure or BAT transplantation on improving PCOS.

In summary, this Research Topic presented an overview of the current advances in the context of adipogenesis and adipocyte energy metabolism. The manuscripts on this topic emphasize that diverse signaling could coordinate adipogenesis and adipocyte metabolism, which can alter systemic insulin resistance and inflammatory response (**Figure 1**). Furthermore, it highlights that pluripotent stem cells isolated from adipose tissue can be applied to cell therapy for a wide range of diseases. Therefore, more detailed studies on the regulatory mechanisms for maintaining energy homeostasis in adipose tissue are warranted in future studies.

**Figure 1:**
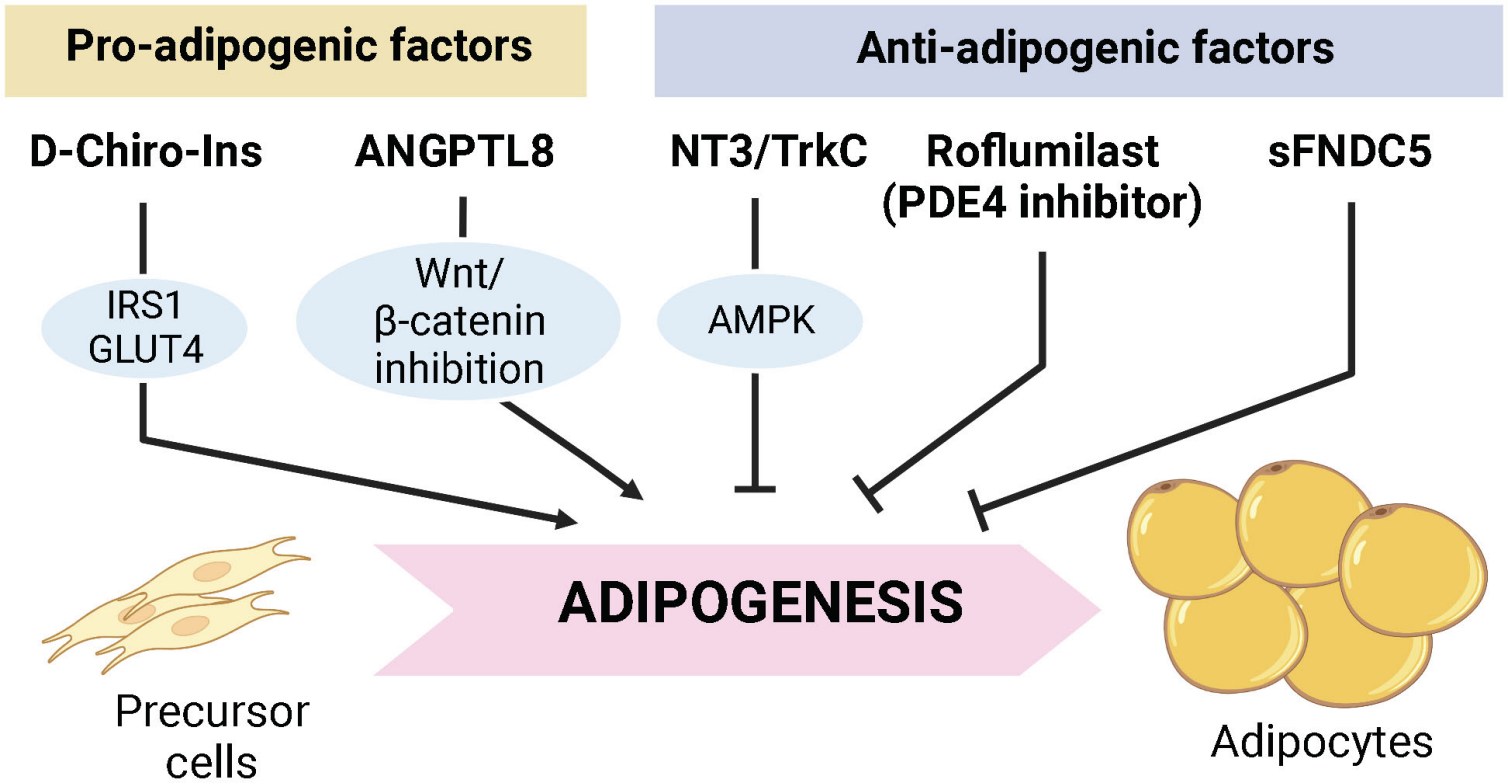
Proposed factors modulating adipogenesis in this research topic. D-Chiro-Ins and ANGPTL8 are suggested to stimulate adipocyte differentiation and lipid deposition. On the other hand, NT3/Trtk3 axis, Roflumilast, and recombinant sFNDC5 are suggested to suppress adipogenesis with increased lipolysis, which might potentially lead to reduced adipose tissue mass.

## Author contributions

All authors listed have made a substantial, direct, and intellectual contribution to the work and approved it for publication.

## Acknowledgments

This work was supported by National Research Foundation of Korea (NRF) grants funded by the Korean government (MSIT) 2021R1C1C2010446 to JYH and National Institute of General Medical Sciences of the National Institutes of Health under Award Number P20GM121334 to DGR. The content is solely the responsibility of the authors and does not necessarily represent the official views of the National Institutes of Health. The figure was created with BioRender.com.

## Conflict of interest

The authors declare that the research was conducted in the absence of any commercial or financial relationships that could be construed as a potential conflict of interest.

## Publisher’s note

All claims expressed in this article are solely those of the authors and do not necessarily represent those of their affiliated organizations, or those of the publisher, the editors and the reviewers. Any product that may be evaluated in this article, or claim that may be made by its manufacturer, is not guaranteed or endorsed by the publisher.
